# Macrophage-Derived Adenosine Deaminase 2 Correlates with M2 Macrophage Phenotype in Triple Negative Breast Cancer

**DOI:** 10.3390/ijms22073764

**Published:** 2021-04-05

**Authors:** Barbara Kutryb-Zajac, Gabriela Harasim, Agata Jedrzejewska, Oliwia Krol, Alicja Braczko, Patrycja Jablonska, Paulina Mierzejewska, Jacek Zielinski, Ewa M. Slominska, Ryszard. T. Smolenski

**Affiliations:** 1 Department of Biochemistry, Medical University of Gdansk, 80-211 Gdansk, Poland; gabriela.harasim@gumed.edu.pl (G.H.); agata.jedrzejewska@gumed.edu.pl (A.J.); oliwia.krol@gumed.edu.pl (O.K.); alicja.braczko@gumed.edu.pl (A.B.); patrycja.jablonska@gumed.edu.pl (P.J.); paulina.mierzejewska@gumed.edu.pl (P.M.); ewa.slominska@gumed.edu.pl (E.M.S.); 2 Department of Surgical Oncology, Medical University of Gdansk, 80-210 Gdansk, Poland; jacek.zielinski@gumed.edu.pl

**Keywords:** adenosine deaminase, breast cancer, TNBC, ADA1, ADA2, macrophages

## Abstract

Several lines of evidence suggest that altered adenosine deaminase (ADA) activity, especially its ADA2 iso-enzyme, is associated with malignant breast cancer (BC) development. Triple-negative breast cancer (TNBC) is currently the most challenging BC subtype due to its metastatic potential and recurrence. Herein, we analyzed the sources of ADA iso-enzymes in TNBC by investigating the effects of cell-to-cell interactions between TNBC cells, macrophages, lymphocytes, and endothelial cells. We also examined the potential relationship between ADA activity and cancer progression in TNBC patients. In vitro analyses demonstrated that the interactions of immune and endothelial cells with MDA-MB-231 triple negative BC cells modulated their extracellular adenosine metabolism pattern. However, they caused an increase in the ADA1 activity, and did not alter ADA2 activity in cancer cells. In turn, the co-culture of MDA-MB-231 cells with THP-1 monocyte/macrophages, Jurkat cells, and human lung microvascular endothelial cells (HULEC) caused the increase in ADA2 activity on THP-1 cells and ADA1 activity on Jurkat cells and HULEC. Clinical sample analysis revealed that TNBC patients had higher plasma ADA2 activities and lower ADA1/ADA2 ratio at advanced stages of cancer development than in the initial stages, while patients with hormone receptor positive, HER2 negative (HR+HER2-), and triple positive (HR+HER2+) breast cancers at the same stages showed opposite trends. TNBC patients also demonstrated positive associations between plasma ADA2 activity and pro-tumor M2 macrophage markers, as well as between ADA1 activity and endothelial dysfunction or inflammatory parameters. The analysis of TNBC patients, at 6 and 12 months following cancer treatment, did not showed significant changes in plasma ADA activities and macrophage polarization markers, which may be the cause of their therapeutic failure. We conclude that alterations in both ADA iso-enzymes can play a role in breast cancer development and progression by the modulation of extracellular adenosine-dependent pathways. Additionally, the changes in ADA2 activity that may contribute to the differentiation of macrophages into unfavorable pro-tumor M2 phenotype deserve special attention in TNBC.

## 1. Introduction

Triple-negative breast cancer (TNBC) is the most metastatic and prone to relapse type of breast cancer (BC). [1] It does not express estrogen receptors (ER), progesterone receptors (PR), and human epidermal growth factor receptors (HER2). The histological diversity of different breast cancer types leads to the acquisition of specific features for their carcinogenesis, microenvironment, response to treatments, and clinical behavior. Because TNBC tumors lack ER, PR, and HER2 expression, they are not sensitive to endocrine therapy or HER2 treatment, and standardized TNBC treatment regimens are still lacking. [2] Therefore, understanding molecular mechanisms underlying TNBC and the development of new TNBC treatment strategies have become an urgent clinical need.

Adenosine deaminase (ADA, E.C.3.5.4.4) is an enzyme that catalyzes the irreversible deamination of either adenosine (Ado) or deoxyadenosine (dAdo). [3] This reaction appears to be one of the rate limiting steps in Ado degradation and hence an important regulator of Ado availability. ADA occurs in two iso-enzymes, ADA1 and ADA2. [4] ADA1 is present in all tissues and the majority of ADA activity is derived from ADA1. Most human cells contain very small amounts of ADA2 and its main source is likely to be the monocyte–macrophage cell system. Interestingly, the ADA2 iso-enzyme is predominant in human serum. Due to low K_m_ value (5.2 × 10^−5^ mol/L) and optimal pH at 7.0–7.5, ADA1 is highly efficient for lower substrate concentration in biological sites where pH is neutral. [5] In turn, ADA2 has a K_m_ value of 200 × 10^−5^ mol/L and an optimum pH of 6.5, making it efficient for deamination higher levels of Ado in slightly acidic environment, for example during hypoxia. [6] It has been speculated that both ADA iso-enzymes can interact with membrane proteins and exist as ecto-enzymes, deaminating substrates in extracellular compartment. [7] The primary role of ADA1 is to eliminate intracellular toxic derivatives of Ado and dAdo and protect the cells from apoptosis. [8] It was shown that activation and proliferation of lymphocytes are inhibited by high levels of Ado. Thus, the absence of active ADA1 as a consequence of inherited mutations results in SCID. [9] Also cell–surface ecto-ADA1 (eADA1) plays a critical role in the regulation of immune response primarily related to T lymphocytes. [8] Furthermore, our recent studies highlighted the importance of enhanced eADA1 in endothelial cell activation, which resulted in the alterations of adenosine-dependent pathways. [10,11] Although the biological roles of ADA1 in the immune system and vascular inflammation are well understood, the function of ADA2 remains enigmatic. It has been found that ADA2 is secreted by monocytes undergoing differentiation into macrophages or dendritic cells. [12] Defects in ADA2 have been linked to impaired macrophage M2 polarization, suggesting its role in redirecting macrophage polarization from the M1 to M2 subtype. [13] Indeed, ADA2 has been recognized as a growth-factor with an autocrine activity that induces monocytes’ proliferation and promotes the differentiation of M2 anti-inflammatory macrophages. [14] There are also indirect evidences of a possible role of ADA2 as growth-factor for endothelial cells. [15] This discovery of the growth factor-like activity of ADA2 suggests that this enzyme could be used as a drug target candidate to modulate the immune responses during inflammation and cancer. In our previous study, we have shown that deoxycoformycin (ADA1 and ADA2 inhibitor) counteracted BC-induced endothelial dysfunction, decreased 4T1 BC cell adhesion and transmigration through the endothelial cell layer, and suppressed migration and invasion of murine and human BC cells.

Several lines of evidence suggest that altered ADA activity is associated with malignant BC progression. [16] It has been shown that both ADA iso-enzymes were elevated in tumor tissues of BC patients correlating with tumor grade, size and lymph node involvement. [17,18] Moreover, some BC patients showed an increase in plasma ADA2 activity. [17] However, these data are sparse and do not take into account histological subtypes of BC. We speculate that TNBC development deserves particular attention in terms of ADA2 activity. The reprogramming of tumor-associated macrophages (TAMs) into the anti-inflammatory M2 phenotype has been recently found in TNBC, while the differentiation of TAMs toward the M1 phenotype has been proposed as a promising therapeutic approach for cancer treatment. [19] However, there is a lack of data on the involvement of ADA iso-enzymes in these processes. In this work we aimed to determine the sources of ADA iso-enzymes in TNBC by investigating the effects of cell-to-cell interactions between TNBC cells, macrophages, lymphocytes, and endothelial cells. We also examined the potential relationship between ADA iso-enzyme activities and TNBC progression in BC patients.

## 2. Results

### 2.1. Immune and Endothelial Cells Modulate ADA1 Activity on the Surface of MDA-MB-231 Triple Negative Breast Cells

**Figure 1:**
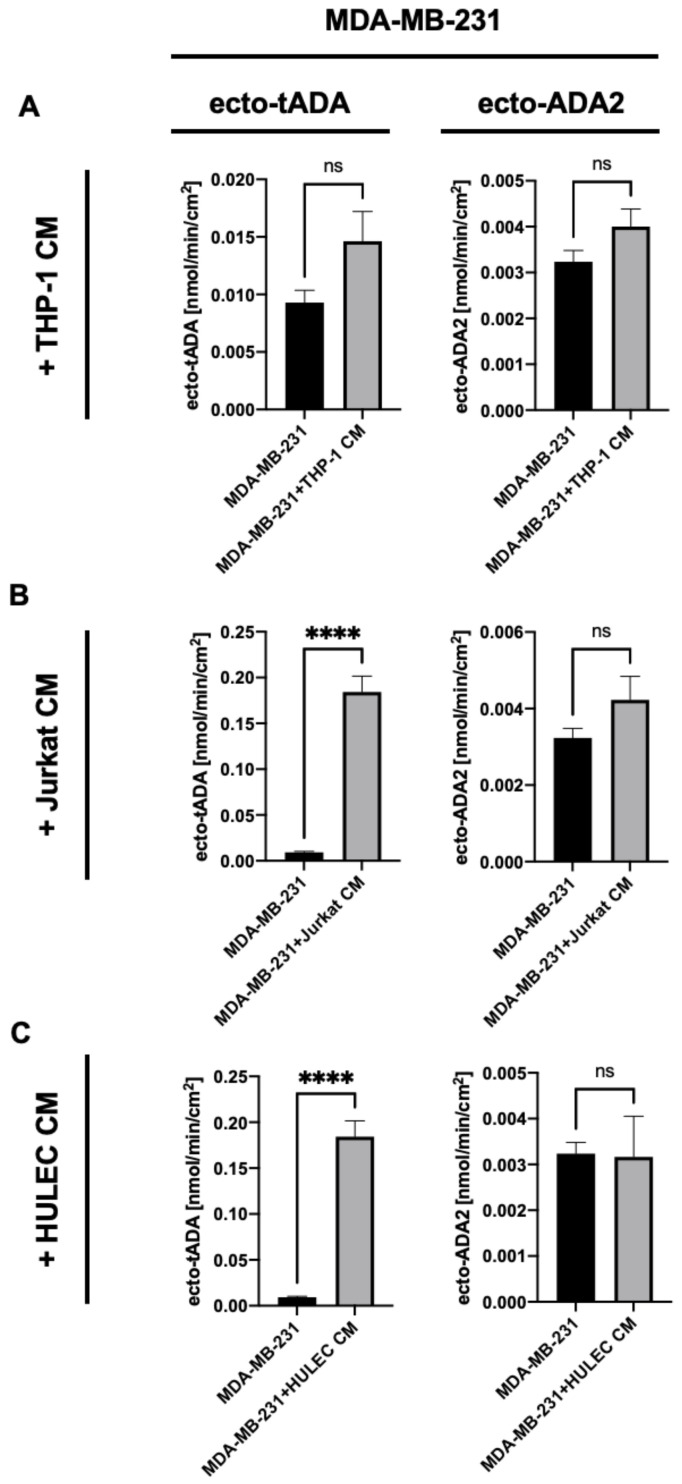
Total cell surface adenosine deamination rate (ecto-tADA) and ADA2 related cell surface adenosine deamination rate (in the presence of ADA1 inhibitor, EHNA) on human triple negative breast cancer cells (MDA-MB-231 cell line) after 48 h incubation with human monocyte/ macrophages medium (THP-1 CM, **A**), Jurkat cell medium (Jurkat CM, **B**), and human microvascular lung endothelial cell medium (HULEC CM, **C**). Results are shown as mean ± SEM, *n* = 6−9, **** *p* < 0.0001 by unpaired *t*-test.

To determine the effects of immune and endothelial cell-released factors on ADA iso-enzyme activities on the surface of breast cancer cells, we incubated human MDA-MB-231 triple negative breast cells with the cell-free medium obtained from the culture of human THP-1 monocytes/macrophages, Jurkat E6.1 lymphocytes or human lung microvascular endothelial cells (HULEC). After 48 h incubation with medium from THP-1 cells, MDA-MB-231 cells have shown no changes in tADA and ADA2 activities on their surface (Figure 1A). In turn, the incubation of MDA-MB-231 cells with Jurkat and HULEC medium led to the increased activity of tADA, without affecting ADA2 (Figure 1B,C).

**Figure 2:**
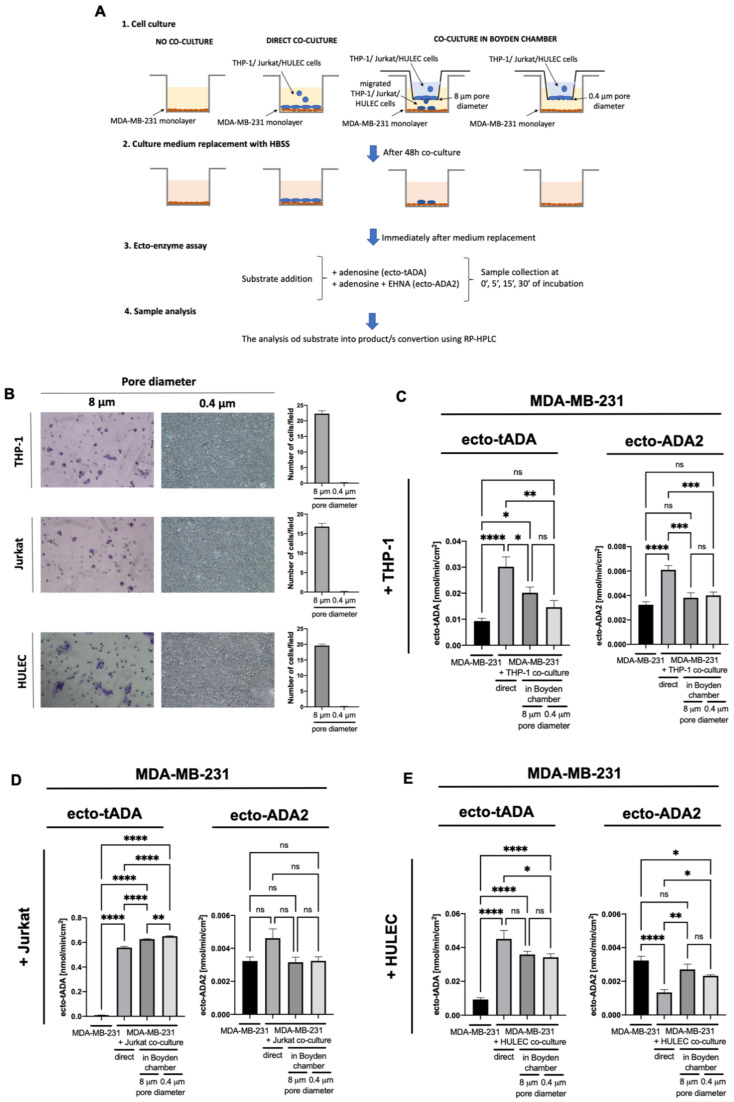
The experimental protocol (**A**), representative images and quantitative analysis of cells migrated via Boyden chambers stained with crystal violet for detection (**B**), total cell surface adenosine deamination (ecto-tADA) and in the presence of ADA1 inhibitor EHNA (ecto-ADA2) on human triple negative breast cancer cells (MDA-MB-231 cell line) after co-culture with human monocytes/macrophages (THP-1, **C**), Jurkat cells (**D**), and human microvascular lung endothelial cells (HULEC, **E**). Results are shown as mean ± SEM, *n* = 6−9, * *p* < 0.05, ** *p* < 0.01, *** *p* < 0.001, **** *p* < 0.0001 by unpaired *t*-test (**B**), one-way ANOVA followed by Holm-Sidak post hoc test (**C**,**D**), and Kruskal-Wallis test followed by Dunn’s post hoc test (**E**).

Then, we analyzed the effects of interactions between immune, endothelial, and cancer cells on ADA iso-enzyme activities in MDA-MB-231 cells. The experimental conditions (Figure 2A) included control cancer cells that were not in co-culture, cancer cells that were directly and indirectly (using Boyden chambers) co-cultured with THP-1, Jurkat, or HULEC cells. To imitate the conditions of immune and endothelial cell migration, we used 8 μm pore size inserts with Boyden chambers. A similar rate of migration was observed for each type of cells used (Figure 2B). To allow the exchange of soluble factors between cells without migration and contact, 0.4 μm pore size inserts were used (Figure 2A,B). Direct co-culture of MDA-MB-231 with THP-1 cells resulted in a significantly higher ecto-tADA and ecto-ADA2 activities (Figure 2C). In turn, the co-culture of these cells in Boyden chamber with 8 μm pore size inserts only increased tADA. In each type of cancer cells co-culture with Jurkat cells, ecto-tADA activity was increased, while ecto-ADA2 was not changed (Figure 2D). Similarly, tADA activity, but not ADA2, was higher after co-culture of MDA-MB-231 with HULEC (Figure 2E).

### 2.2. MDA-MB-231 Triple Negative Breast Cancer Cells Stimulate ADA2 Activity on the Surface of Monocytes/Macrophages and ADA1 Activity on Lymphocytes and Endothelial Cells 

**Figure 3:**
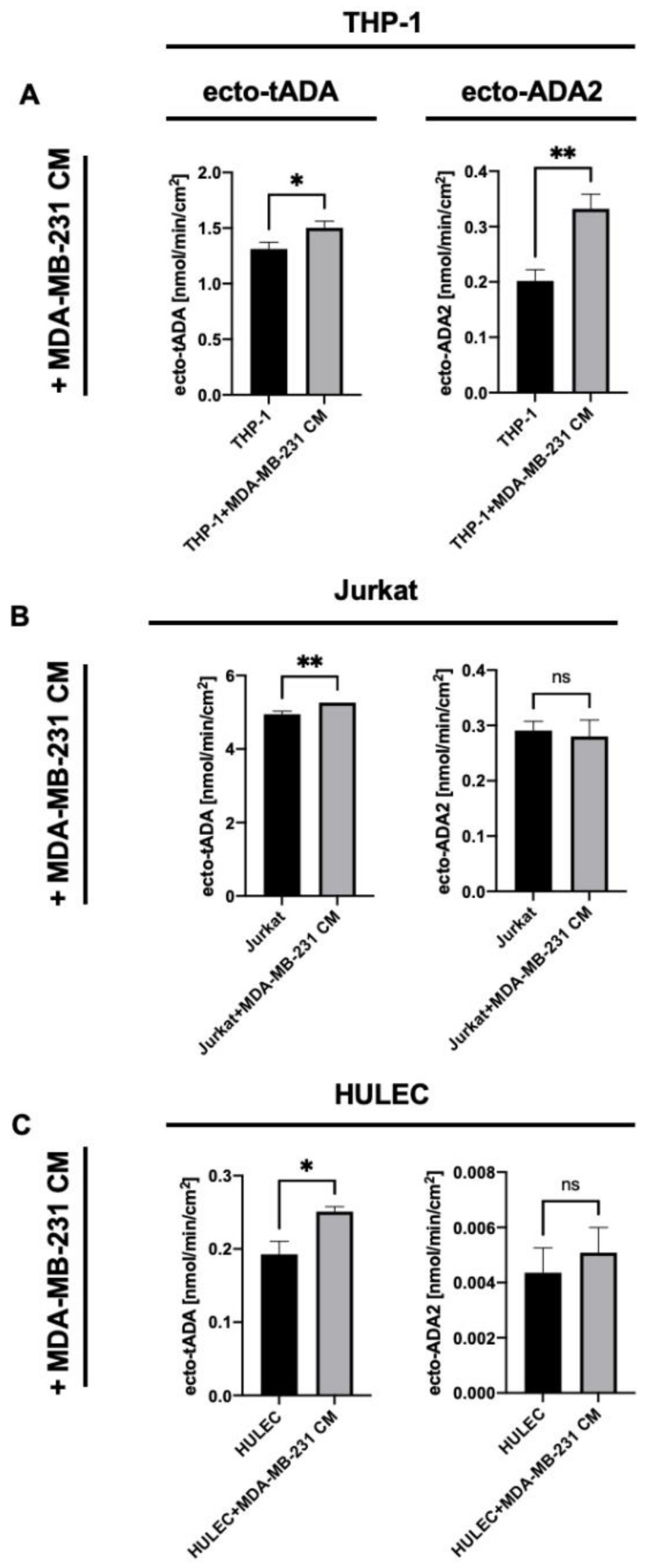
Total cell surface adenosine deamination rate (ecto-tADA) and ADA2 related cell surface adenosine deamination rate (in the presence of ADA1 inhibitor, EHNA) on human monocyte/macrophages (THP-1, **A**), Jurkat cells (**B**), and human microvascular lung endothelial cells (HULEC, **C**) after 48 h incubation with human triple negative breast cancer cell medium (MDA-MB-231 CM). Results are shown as mean ± SEM, *n* = 6−9, * *p* < 0.05, ** *p* < 0.01 by Mann-Whitney test.

Next, we incubated each type of immune cells and HULEC with the cell-free medium obtained from MDA-MB-231 culture. This experimental setup allowed to check the effects of cancer cell-released factors on ecto-ADA iso-enzyme pattern in immune and endothelial cells. THP-1 cells revealed increased activities of both, ecto-tADA, and ecto-ADA2 after incubation with cancer cell media (Figure 3A). Whereas, in Jurkat cells and HULEC only tADA activity was elevated (Figure 3B,C).

**Figure 4:**
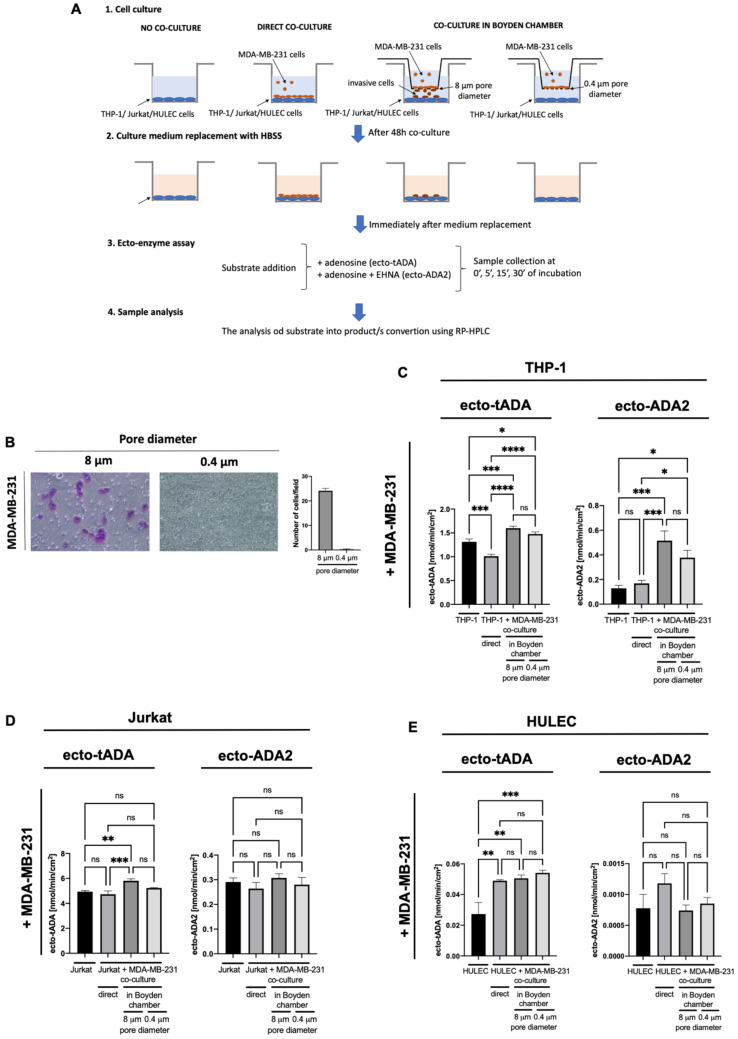
The experimental protocol (**A**), representative images and quantitative analysis of cells migrated via Boyden chambers stained with crystal violet for detection (**B**), total cell surface adenosine deamination rate (ecto-tADA) and in the presence of ADA1 inhibitor EHNA (ecto-ADA2) on human monocyte/macrophages (THP-1, **C**), Jurkat cells (**D**), and human microvascular lung endothelial cells (HULEC, **E**) after co-culture with human triple negative breast cancer cells (MDA-MB-231 cell line). Results are shown as mean ± SEM, *n* = 6−9, * *p* < 0.05, ** *p* < 0.01, *** *p* < 0.001, **** *p* < 0.0001 by unpaired *t*-test (**B**), Kruskal-Wallis test followed by Dunn’s post hoc test (**C**), and one-way ANOVA followed by Holm-Sidak post hoc test (**D**,**E**).

When analyzing the immune and endothelial cell co-culture with MDA-MB-231, as was shown on Figure 4A, we observed the augmented activities of ecto-tADA and ecto-ADA2 on THP-1 cells using Boyden chambers with both 8 μm and 0.4 μm pore size inserts (Figure 4B,C). Invaded cancer cells that migated through 8 μm pores also caused the increase in tADA activity on Jurkat lymphocytes (Figure 4D). Each type of HULEC co-culture with MDA-MB-231 cells, increased only ecto-tADA activity (Figure 4E).

### 2.3. Plasma ADA Iso-Enzyme Pattern Is Adversely Deregulated in Different Subtypes Breast Cancers 

In the next stage, we analyzed patients with breast cancer, characterized as hormone receptor (HR) positive and HER2 positive (HR+HER2+), HR positive and HER2 negative (HR+HER2-), and HR negative, HER2 negative (triple negative, TNBC). Breast cancer subgroups as well as healthy controls were characterized in terms of biochemical and endothelial function parameters (Table 1). TNBC patients had significantly higher concentrations of asymmetric dimethylarginine (ADMA), one of the endogenous inhibitors of nitric oxide synthase (NOS), compared to the control (Table 1). Breast cancer patients were also characterized based on the cancer histological type, involved lymph nodes, proliferation marker Ki-67, blood morphology, electrolytes concentration, and coagulation parameters (Table 2, Tables S1–S3). There was a significantly higher % of Ki-67 among TNBC patients compared to the HR+HER2- group. On the other hand, the HR+HER2- patients had a lower Ki-67 compared to the HR+ HER2+ group (Table 2). When we analyzed patients with different subtypes of breast cancer, according to its stage, significantly higher lymph nodes involvement was noted at stage III as compared to previous stages in each of the studied groups (Tables S1–S3).

**Figure 5:**
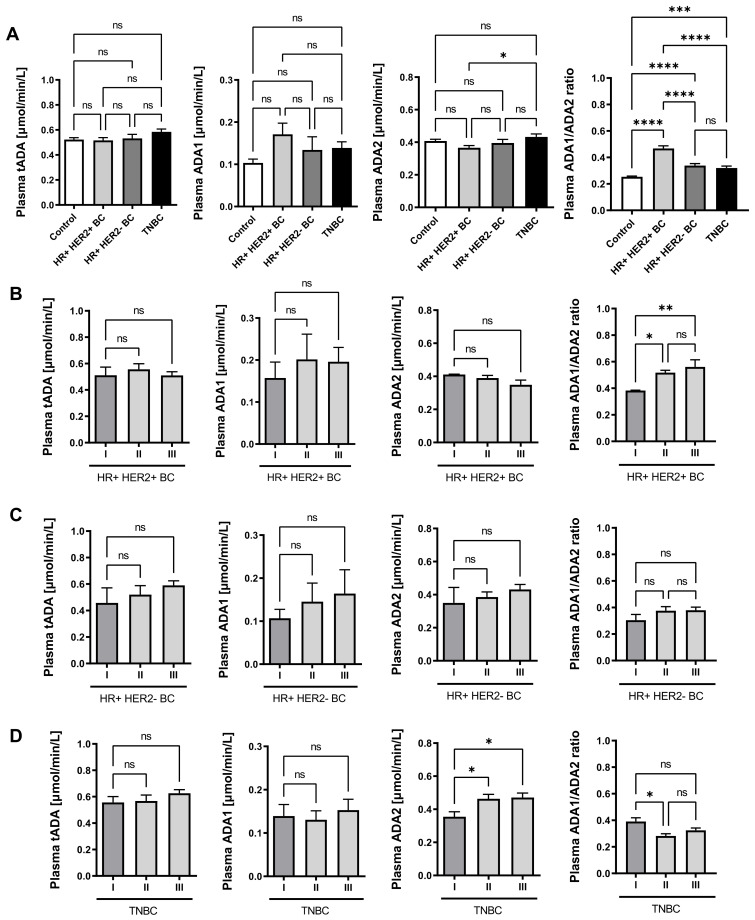
Plasma adenosine deaminase (ADA) activity in breast cancer patients. The activity of total ADA (tADA), ADA1, and ADA2 in healthy controls (*n* = 18); estrogen (ER) and progesterone (PR) receptor positive, HER2 positive (HR+ HER2+ BC, *n* = 12); ER and PR positive, HER2 negative (HR+ HER2- BC, *n* = 16); and triple negative (TNBC, *n* = 19) breast cancer patients (**A**); in HR+ HER2+ BC/ HR+ HER2- BC/ TNBC patients with different stages of cancer development (I stage: *n* = 3/7/7; II stage: *n* = 6/4/5; III stage: *n* = 3/4/7) (**B**). Results are shown as mean ± SEM, * *p* < 0.05, ** *p* < 0.01, *** *p* < 0.0001, **** *p* < 0.00001 by one-way ANOVA followed by Holm-Sidak post hoc test (**A**, **C,** and **D**) and Kruskal-Wallis test followed by Dunn’s post hoc test (**B**).

Then, plasma activities of total ADA and its isoenzymes were determined in breast cancer patients. There were no significant differences in total ADA activity (tADA) in plasma between the studied groups of patients. Only a trend towards higher ADA1 activity in plasma of breast cancer patients compared to healthy controls was noted. However, a significantly higher ADA2 activity in the plasma of TNBC patients was demonstrated compared to HR+HER2+ patients (Figure 5A). HR+HER2+ patients also revealed the highest ratio of plasma ADA1/ADA2 activity (Figure 5B). Moreover, ADA1/ADA2 ratio grew with cancer stage in HR+HER2+ BC (Figure 5B). A similar pattern of plasma ADA iso-enzyme activities was maintained in HR+HER2- BC according to the rate of cancer development, but only a tendency in increased ADA1 activity and ADA1/ADA2 ratio was observed (Figure 5C). Interestingly, we noted higher ADA2 activity as well as lower ADA1/ADA2 ration in the plasma of stage II and III TNBC patients compared to stage I patients (Figure 5D).

### 2.4. Plasma ADA2 Activity Correlates with M2 Macrophage Phenotype in Triple Negative Breast Cancer

**Figure 6:**
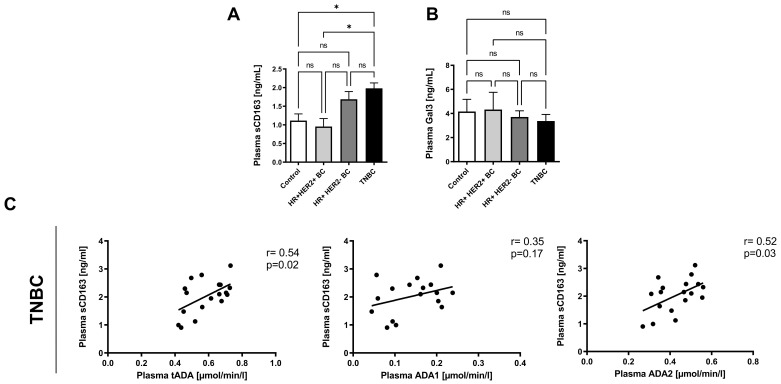
Plasma macrophage polarization markers in breast cancer patients. Plasma concentration of soluble CD163 protein (sCD163, **A**) and galectin 3 (Gal3, **B**) in healthy controls (*n* = 18), HR+ HER2+ BC (*n* = 12), HR+ HER2- BC (*n* = 16), and triple negative (TNBC, *n* = 19) breast cancer patients (**C**). Correlation plots of plasma sCD163 with tADA, ADA1, and ADA2 activities in TNBC (**D**). Results are shown as mean ± SEM, * *p* < 0.05 by one-way ANOVA followed by Holm-Sidak post hoc test (**A**), Kruskal-Wallis test followed by Dunn’s post hoc test (**B**) and as Pearson correlation coefficients with corresponded *p*-value (**C**).

Next, we analyzed the relation between macrophage polarization profile and plasma ADA iso-enzyme activities in breast cancer patients. The concentrations of soluble CD163, a marker of macrophages M2 and galectin 3 (Gal3), rather related to M1 macrophages, in the plasma of healthy controls and breast cancer patients were also determined. There were no significant differences in Gal3 concentration between the studied groups of patients. Interestingly, a significantly higher concentration of sCD163 in the plasma of TNBC patients was noted compared to HR+HER2+ patients and healthy controls (Figure 6A,B). A positive correlations of the plasma sCD163 with the activities of tADA and ADA2 were also demonstrated (Figure 6C). In turn, the concentration of plasma galectin-3 tended to the negative correlation with both ADA iso-enzymes in TNBC (Table 3). The positive correlations were observed between plasma ADA1 activity, hsCRP, ADMA concentration and the ratio of ADMA/L-arginine. Moreover, the activities of tADA and ADA2 positively correlated with plasma ADMA concentrations in TNBC patients.

**Figure 7:**
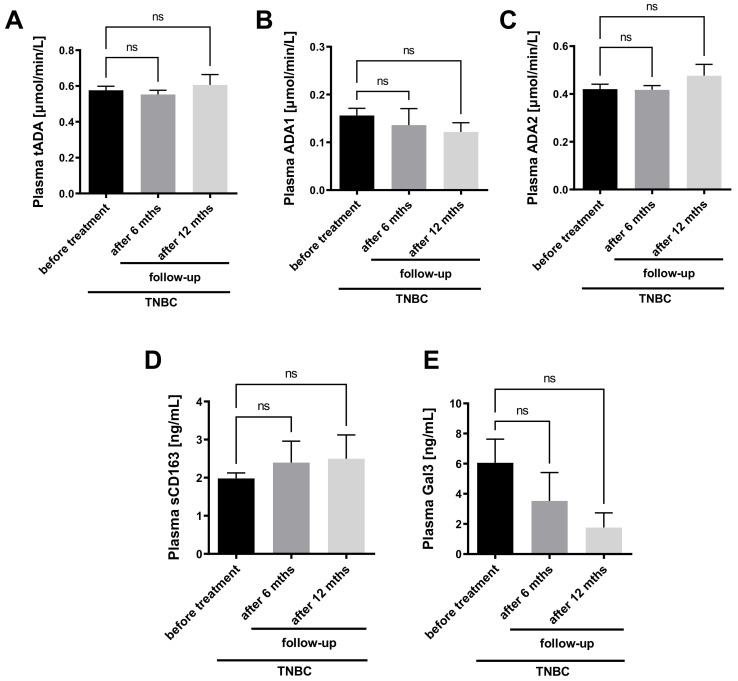
Plasma adenosine deaminase activity and macrophage polarization markers in TNBC patients before and after cancer treatment. The activity of plasma total ADA (tADA, **A**), ADA1 (**B**), ADA2 (**C**), and concentration of plasma soluble CD163 (sCD163, **D**) and galectin-3 (Gal3, **E**) in triple negative breast cancer (TNBC) patients before (*n* = 19), 6-months (6 mths, *n* = 6) and 12-months (12 mths, *n* = 4) after surgical intervention followed by chemotherapy. Results are shown as mean ± SEM and analyzed by Kruskal-Wallis test followed by Dunn’s post hoc test (**A**,**B**,**E**) and one-way ANOVA followed by Holm-Sidak post hoc test (**C**,**D**).

Furthermore, the activities of tADA, ADA1 and ADA2 were measured in the plasma of TNBC patients before and after the treatment. There were no significant differences in tADA, ADA1, and ADA2 activities between patients 6- and 12-months after surgical intervention and subsequent standard chemotherapy protocol (doxorubicin and cyclophosphamide followed by paclitaxel) compared to patients before the treatment (Figure 7A–C). There was also only a trend for a higher concentration of soluble CD163 (Figure 7D) and a lower concentration of Gal3 in patients after the treatment compared to those before (Figure 7E).

## 3. Discussion

The major finding of this study is the demonstration that monocytes and macrophages are a major source of the increased ADA2 activity in the response to the stimulation with TNBC cells. In turn, TNBC cell-activated endothelial cells and lymphocytes were sources of increased ADA1 activity. Plasma ADA2 activity was higher at advanced stages of TNBC development than in the initial stages and positively correlated with M2 macrophage phenotype. In turn, plasma ADA1 activity correlated with endothelial dysfunction and inflammatory parameters. Interestingly, only TNBC patients with the grades II and III exhibited decreased plasma ADA1/ADA2 ratio, while HR+HER2+ and HR+HER2- patients at the same stages showed an opposite trend. We suggest that both ADA iso-enzymes could be valuable therapeutic targets in the breast cancer. Especially in TNBC, the inhibition of ADA2 that can redirect macrophages into the preferred anti-tumor M1 phenotype deserves attention, while the suppression of ADA1 activity may be of importance to protect the endothelium and reduce metastasis.

Adenosine is a critical regulatory autocrine and paracrine factor that accumulates in the tumor microenvironment. [20] It acts through four types of G-coupled adenosine receptors (ARs): A1, A2a, A2b, and A3. [21] It is believed that AR activation on immune cells regulates inflammatory functions and switches immune surveillance and host defense to promotion of cancer transformation and growth. [22] Several lines of evidence showed that adenosine pathways also regulate cancer growth and dissemination by interfering with cancer cell proliferation, apoptosis, and metastasis. This suggests that, depending on AR subtype engaged on neoplastic cells, it has distinct effects. In most cancers, the stimulation of A1, A2a, and A2b receptors induces cancer cell proliferation, while A3 receptor activation limits this process. Other studies revealed that adenosine via A2a, A2b, and A3 receptors activates tumor cell apoptosis through caspase-dependent or independent modes. [22] In our previous study, we have demonstrated that AR stimulation (A3 and partly A2a) decreased adhesion of murine 4T1 breast cancer cells to the H5V endothelial cell layer, while A2a receptor activation decreased 4T1 cell invasion. [23] We have assumed that these AR-mediated effects could be one of the anti-tumor properties of ADA inhibition revealed by dCF in our murine 4T1 breast cancer model. On the other hand, it might be a result of endothelial protection exerted by AR mechanisms. Recently, we also demonstrated that ADA inhibition exhibited positive effects on endothelial cells via AR stimulation, including decreased expression of adhesion molecules and improvement of endothelial barrier function. [11,23]

The dominant pathway leading to high adenosine concentration in the tumor microenvironment is the rapid extracellular phosphohydrolysis of ATP by ecto-nucleotidases, CD39, and CD73. [24,25] The termination of adenosine signaling in extracellular space is dependent on two mechanisms, adenosine uptake via nucleoside transporters and cell surface metabolism to inosine by ADA. [26,27] It has been shown that many cancers, including breast cancer, are associated with elevated ADA activity. [18,23,28,29,30] In our previous study, we have demonstrated that immune cells, endothelial cells, and to a lesser extent cancer cells are sources of ADA1 iso-enzyme activity in breast cancer, while circulating monocytes and most probably tumor-associated macrophages are a dominant source of intra- and extracellular ADA2 activity. [23]

In this work, for the first time we examined the impact of triple negative cancer cell interactions with immune and endothelial cells on the cell–surface ADA activities. We have shown that ecto-tADA activity was increased on MDA-MB-231 cancer cells after the incubation with medium obtained from immune (THP-1 and Jurkat) and endothelial (HULEC) cells, while their effect on ecto-ADA2 activity was not changed. Such metabolic pattern can be beneficial for cancer cells as it removes adenosine from their immediate environment and thus protects them against adenosine-dependent apoptosis. [31,32] It also suggests that endothelial and immune cells can release soluble factors, for example interleukins or growth factors, which can stimulate ecto-ADA1 activity on cancer cells. Similar observations of the increase in ecto-ADA activity have been found in co-culture experiments. However, in both direct and indirect co-culture conditions, lymphocytes and endothelial cells caused a greater increase in eADA1 activity on MDA-MB-231 cancer cells than incubation with the medium from these cells. As in direct co-culture, and co-culture in a Boyden chamber with 8 µm pore size, the acquired eADA1 activity may come from attached or migrated cells, the significant increase in eADA1 on cancer cells using 0.4 µm pore size inserts is puzzling. Previously, we have shown that lymphocytes and endothelial cells can release eADA1 protein into the extracellular space under pathological stimulation. [11] We assume that the co-culture of Jurkat and HULEC with cancer cells can also induce them to secrete the enzyme, and that this soluble eADA1 can be attached on the surface of adjacent cells that have ADA-binding proteins (eg. CD26 or adenosine receptors), in this case on cancer cells. However, the processes of eADA1 exchange between cells required further studies.

On the other hand, the treatment of immune and endothelial cells with cancer cell medium, increased ecto-ADA2 activity on THP-1 monocytes/macrophages and ecto-ADA1 on Jurkat and HULEC. Ecto-ADA2 activity particularly increased on the surface of THP-1 cells in a co-culture with cancer cells using 8 µm size pore inserts. This demonstrates that migrated, and hence the most invasive, TNBC cells significantly contributed to the increase of ADA2 on monocytes and macrophages. This may be especially important for tumor progression, as ADA2, on the one hand, effectively degrades adenosine in a hypoxic tumor microenvironment, but also acts as a growth factor for anti-inflammatory M2 macrophages. [6,14] Indeed, the plasma activity of ADA2 positively correlated with M2 macrophage marker, sCD163 in TNBC patients. In turn, galectin-3, which can be rather related to M1 macrophage activity tended to negatively correlate with both ADA1 and ADA2 in these patients. [33] Reprogramming the M2 macrophage phenotype toward the pro-inflammatory M1 phenotype has been recognized as a promising therapeutic approach for the treatment of breast cancer. [34,35] Although, the analysis of TNBC patients 6- and 12-months following cancer treatment and subsequent chemotherapy did not show changes in the plasma profile of ADA activities and macrophage polarization markers that can provide an unfavorable phenotype, which may lead to cancer recurrence. Although the exact effect of currently used breast cancer therapies on ADA activities require further studies, we believe that novel strategies, which based on modulating ADA activities may be important in enhancing their effectiveness.

The increase in ecto-ADA1 activity on Jurkat T lymphocytes and HULEC endothelial cells can also be viewed as a negative factor that promotes cancer progression. Although the infiltration of T lymphocytes, particularly CD8^+^ T cells (TILs) is usually associated with a favorable prognosis and may predict outcome of therapies with drugs that block immune inhibitory receptors, it has been reported that TNBC shows the highest incidence of lymphocyte predominance. [36] It has been shown that increased percentages of TILs are associated with enhanced immune-suppressive functions by various cell populations such as stromal cells, myeloid cells, or regulatory T cells. [37] Moreover, increased activity of ADA2 derived from TAMs can induce T cell-dependent further differentiation of monocytes into macrophages and stimulate macrophage proliferation activating them towards the M2 phenotype. [12,38]

We have also shown a significant effect of MDA-MB-231 cells on ecto-ADA1 activity in HULEC during the stimulation with cancer cell medium as well as in co-culture. Interestingly, the highest ecto-ADA1 activity on endothelial cells was observed when the cells were co-cultured using a 0.4 μm-sized pore, which prevented the migration of cancer cells via the membrane. This underlines the impact of factors secreted by cancer cells on the increase of the cell-surface ADA activity in endothelial cells. As breast cancer cells are a minor source of soluble ADA [23], we exclude the possibility of anchoring cancer cell-derived ADA on endothelial cells. However, it has been found that vascular endothelial growth factor (VEGF) itself is abundantly secreted by breast cancer cells in order to promote differentiation and an aggressive phenotype. [39,40] VEGF is the most important pro-angiogenesis factor and highly dysregulated in TNBC. [41] Specifically, VEGF binds to its receptor on the surface of endothelial cells to affect the tumor neovascularization. VEGF can also induce the adhesion and migration of MDA-MB-231 cells when co-cultured with endothelial cells. [42] Moreover, VEGF is a potent inducer of endothelial cell inflammation. It has been showed that it affected on vascular permeability that was dependent on the synthesis of platelet-activating factor by endothelial cells. [43] We have found previously that endothelial-derived ecto-ADA1 can be a useful biomarker of endothelial cell activation. [10,11] Moreover, by the removing of extracellular adenosine, increased endothelial ecto-ADA1 activity led to the adverse phenotype of endothelial cells that may promote cancer progression and metastasis. [11,23] In this work we have also found that plasma ADA1 activity positively correlated with the markers of endothelial dysfunction and inflammation in breast cancer patients, highlighting the association of adenosine catabolism with endothelial cell function in these patients.

## 4. Conclusions

Significant alterations in both ADA iso-enzyme activities (ADA1 and ADA2) have been found as a result of TNBC cell interactions with macrophages, lymphocytes, and endothelial cells in vitro providing an unfavorable phenotype related to cancer progression. The patients with TNBC revealed higher plasma ADA2 activities at advanced stages of cancer development than in the initial stages and exhibited low ADA1/ADA2 ratio, while patients with HR+HER2+ and HR+HER2- breast cancers at the same stages showed opposite trends. In TNBC, we also found strong positive associations between plasma ADA2 activity and pro-tumor M2 macrophage markers as well as between ADA1 activity and endothelial dysfunction or inflammation parameters that may prone these patients to faster progression of the disease due to adenosine-dependent mechanisms. These results suggest that both ADA1 and ADA2 could be potential targets for breast cancer therapy. Especially for TNBC, the inhibition of ADA2 that can redirect tumor associated macrophages into a preferred anti-tumor M1 phenotype deserves special attention. 

## 5. Materials and Methods

### 5.1. Patients

The study was performed based on the standards of the Declaration of Helsinki and it was approved by the local ethical committee. Informed consent has been obtained from the patients. The study included healthy controls (*n* = 18, females, mean age: 43, median age: 41); 12 patients with hormone receptor positive, human epidermal growth factor receptor 2 positive breast cancer (HR+HER2+ BC, mean age: 54, median age: 52); 16 patients with HR+HER2- BC (mean age: 58, median age: 60); and 19 patients that were diagnosed with triple negative breast cancer (TNBC, mean age: 56, median age: 59). In addition, the samples from TNBC patients were examined 6 months after surgical intervention, when all patients had completed chemotherapy according to the following protocol: 4 AC cycles (cyclophosphamide and doxorubicin) and 12 cycles of paclitaxel (6 mths, *n* = 6), as well as 12months after surgical treatment and subsequent chemotherapy, which was finished 6 months after the surgery (12 mths, *n* = 4). Among treated patients, one of them had a recurrence of the cancer, while the others remain in remission. Plasma were obtained from peripheral blood collected at fasting state into EDTA and citrate tubes, while serum from clotted blood without the addition of an anticoagulant after centrifugation at 1200× *g*, 5 min, 21 °C.

### 5.2. Determination of the Plasma Activities of ADA Iso-Enzymes 

The activities of soluble total ADA and ADA2 in plasma were determined using a previously published protocol. [44] Briefly, EDTA plasma (49 μL), warmed to 37 °C was incubated with 20 μM adenosine (final concentration) in the presence of 5 μM erythro-9-(2-hydroxy-3-nonyl)adenine (EHNA, ADA1 inhibitor). After 30 min, plasma samples were deproteinized with 1.3 M HClO4 (ratio 1:1) and centrifuged (20,800× *g*, 4 °C, 15 min). The supernatant was brought to pH 6.0–6.5 using 3 M K3PO4 and analyzed with HPLC according to the method described earlier. [45,46] ADA1 activity was calculated from the difference between tADA and ADA2 activities.

### 5.3. Detemination of Plasma Markers of Macrophage Polarization, Clinical Biochemistry Panel, Blood Morphology, Electrolites and Coagulation Parameters in Patients 

Concentration of soluble CD163 and galectin-3 in human plasma were measured using enzyme-linked immunosorbent assay kits according to the manufacturer’s protocols. HsCRP, albumin, total protein, alkaline phosphatase, lactate dehydrogenase, calcium, magnesium, phosphorus, aspartate transaminase, alanine transaminase, urea, and lipid profile parameters were measured in patient serum or plasma (if appropriate) using an Automated Photometer (ERBA XL-180, Mannheim, Germany) and specific ERBA kits according to manufacturer’s instructions. Parameters of the blood morphology, electrolytes, coagulation system, glycemia were measured in patients using standard methods.

### 5.4. Determination of Serum Amino Acid Metabolites 

To evaluate the serum amino acids, as well as l-arginine analogs, an aliquot of serum (50 µL) were extracted with acetonitrile (ratio 1:2.4) and centrifuged (20,800× *g*/10 min/4 °C). Supernatant were collected and freeze dried. The obtained precipitate was dissolved in water at a volume equal to the initial serum volume. Amino acids and derivatives concentration were determined using high performance liquid chromatography–mass spectrometry (LC/MS), as previously described. [10]

### 5.5. Cell Culture 

Human THP-1 monocytes/macrophages and Jurkat E6.1 cells were obtained from ECACC (Salisbury, UK). Human lung microvascular endothelial cells (HULEC-5a) were obtained from ATCC (Manassas, VA, USA). Human triple negative breast cancer cells (MDA-MB-231) were kindly provided by Dr. Patrycja Koszalka (Department of Cell Biology, Faculty of Medical Biotechnology, Medical University of Gdansk, Poland). THP-1 and Jurkat E6.1 were cultured in RPMI 1640 medium (Merck, Darmstadt, Germany) supplemented with 10% fetal bovine serum (FBS; Gibco, Thermofischer, Waltham, MA, USA) and 1% Penicillin-Streptomycin (Merck, Germany) at 37 °C, 5% CO_2_. HULEC were cultured in MCDB131 medium (ATCC, Manassas, VA, USA) supplemented with 10% FBS (Gibco, Thermofischer, Waltham, MA, USA), 10 ng/mL Epidermal Growth Factor (Merck, Darmstadt Germany), 1 µg/mL Hydrocortisone (Merck, Germany), 10 mM L-glutamine (Merck, Darmstadt Germany), and 1% Penicillin-Streptomycin (Merck, Darmstadt Germany) at 37 °C, 5% CO_2_. MDA-MB-231 were cultured in Dubelecco’s modified Eagle’s medium (DMEM, 4.5 g/L glucose, Merck, Darmstadt Germany) supplemented with 10% FBS (Gibco, Thermofischer USA), 2 mM L-glutamine (Merck, Darmstadt Germany), 1 mM sodium pyruvate (Merck, Darmstadt Germany), and 1% penicillin/streptomycin (*v*/*v*, Merck, Darmstadt, Germany) at 37 °C, 5% CO_2_.

### 5.6. Media Transfer

HULEC-5a and MDA-MB-231 were seeded on 24-well plates at a density of 5 × 10^5^ cells/well (HULEC-5a) and 8 × 10^5^ cells/well (MDA-MB-231) and at 80–90% confluency cells were used for experiments in a total volume of 1 mL of FBS-free media. THP-1 and Jurkat E6.1 cells were plated in 24-well cell culture plate at a density 1 × 10^6^ per well in a total volume of 1 mL FBS-free media and coated on the plate by centrifugation (160× *g* for 5 min, 37 °C). After 24 h, cell media from the HULEC-5a, THP-1 and Jurkat E6.1 cells were harvested, centrifuged (160× *g* for 5 min, 37 °C), and used for the incubation with MDA-MB-231 cell monolayers on 24-well plates. As a control, we used FBS-free media dedicated for HULEC-5a, THP-1 and Jurkat E6.1 cell culture. After 48 h incubation, adherent cells were directly washed with Hanks Balanced Salt Solution (HBSS, Merck, Darmstadt, Germany), while adherent cells were centrifuged (160× *g* for 5 min, 37 °C) and then washed with HBSS. Then, cells were used for the determination of cell surface tADA and ADA2 activities in a total volume of 1 mL of HBSS. Accordingly, after 24 h culture of MDA-MB-231 cells on 24-well plates, medium was harvested, centrifuged (160× *g* for 5 min, 37 °C), and used for the incubation with HULEC-5a, THP-1, and Jurkat E6.1 cells. After 48 h incubation, MDA-MB-231 cells were directly washed with HBSS and used for the determination of cell surface tADA and ADA2 activities in a total volume of 1 mL of HBSS.

### 5.7. Co-Culture Conditions

For the co-culture experiments, MDA-MB-231 cells were seeded on 24-well companion plates (BD Falcon, San Jose, CA, USA) at a density of 8 × 10^5^ cells/well in 1 mL of FBS-free media, while HULEC-5a, THP-1, and Jurkat E6.1 cells were seeded on PET 8 and 0.4 μm pore diameter inserts (BD Falcon, USA) at a density of 3 × 10^5^ cells/insert (HULEC-5a) and 0.5 × 10^6^ cells/insert (THP-1 and Jurkat E6.1) in a total volume of 100 μL of FBS-free media. The cells were cultured separately for 24 h to establish attachment. After 24 h, the inserts with HULEC-5a, THP-1, and Jurkat E6.1 cells were transferred into the wells of 24-well companion plate with MDA-MB-231 cell monolayers to create the hanging co-culture setup. For the direct co-culture, HULEC-5a, THP-1, and Jurkat E6.1 cells were seeded at a density of 3 × 10^5^ cells/well (HULEC-5a) and 0.5 × 10^6^ cells/well (THP-1 and Jurkat E6.1) on the surface of MDA-MB-231 cell monolayers. After 48 h of incubation in co-culture, the inserts and cell medium were removed and MDA-MB-231 cells were washed with HBSS and used for the determination of cell surface tADA and ADA2 activities in a total volume of 1 mL of HBSS.

In a different set of experiments, MDA-MB-231 cells were seeded on PET 8 and 0.4 μm pore diameter inserts (BD Falcon, USA) at a density of 4 × 10^5^ cells/insert in a total volume of 100 μL of FBS-free media. HULEC-5a, THP-1, and Jurkat E6.1 cells were seeded on the bottom wells at a density of 5 × 10^5^ cells/well (HULEC) and 1 × 10^6^ cells/well (THP-1 and Jurkat E6.1) in a total volume of 1 mL of FBS-free media. After 24 h, the inserts with MDA-MB-231 cells were moved into the wells with HULEC monolayers or THP-1 and Jurkat E6.1 cells (previously coated on the plates by centrifugation at 160× *g* for 5 min, 37 °C). For the direct co-culture, MDA-MB-231 cells were seeded at a density of 4 × 10^5^ cells/well on the surface of HULEC-5a, THP-1, and Jurkat E6.1 cells. After 48 h of co-culture, the inserts were removed and HULEC-5a cells were directly washed with HBSS, while plates with THP-1 and Jurkat E6.1 cells were centrifuged, the medium was removed and cells were used for the determination of cell surface tADA and ADA2 activities in a total volume of 1 mL of HBSS.

### 5.8. The Analysis of Cell Migration Abilities

HULEC-5a, THP-1 and Jurkat E6.1 and MDA-MB-231 cells were seeded in the upper chamber of cell culture inserts. Then cells were incubated in serum-free medium for 24 h. After the incubation, cells remaining in the upper chamber were removed and the cells that had invaded into the lower chamber were fixed with methanol for 5 min and stained with crystal violet for 15 min at room temperature. Ten fields per insert were imaged and scored using Zeiss inverted microscope.

### 5.9. Determination of the Activities of ADA Iso-Enzymes in Human Cell Lines

To measure cell–surface total ADA activity, cells were incubated in 1 mL of HBSS at 24-well plates with 50 μM adenosine in the presence of 1 μM S-(4-Nitrobenzyl)-6-thioinosine (NBTI), nucleoside transporter inhibitor. Samples were collected after 0, 5, 15, and 30 min of incubation at 37 °C and analysed using HPLC as described earlier. [10] The rate of adenosine deamination was calculated from a linear phase of the reaction and expressed per the culture area as nmol/min/cm^2^. Cell surface ADA2 activity was measured as described above for total ADA, but in the presence of 5 μM erythro-9-(2-hydroxy-3-nonyl)adenine (EHNA, ADA1 inhibitor).

### 5.10. Statistical Analysis

Statistical analysis was performed using InStat software (GraphPad, San Diego, CA, USA). Comparisons of mean values between groups were evaluated by one-way analysis of variance (ANOVA) followed by Holm–Sidak post hoc test, Kruskal-Wallis test followed by Dunn’s post hoc test, unpaired Student’s *t* test, or Mann–Whitney *U* test, after the assessment of normality. The normality was assessed using the Kolmogorov–Smirnov test (when *n* = 5), Shapiro–Wilk test (when *n* = 7), and the D’Agostino and Pearson Omnibus (when *n* ≥ 8) normality tests. The exact value of *n* was provided for each type of experiments. Statistical significance was assumed at *p* < 0.05. Error bars indicated the standard error of the mean (SEM) unless otherwise described in the figure legend.

## Figures and Tables

**Table 1:** Clinical biochemistry profile and endothelial function parameters in controls and breast cancer patients.

Parameter	Control (*n* = 18)	HR+ HER2+ (*n* = 12)	HR+ HER2- (*n* = 16)	TNBC (*n* = 19)
CRPhs, mg/dL	0.37 ± 0.05	0.34 ± 0.02	0.44 ± 0.04	0.38 ± 0.08
LDL, mg/dL	109 ± 10.2	120 ± 21.8	105 ± 6.57	130 ± 17
HDL, mg/dL	65.4 ± 5.82	54.6 ± 5.82	55.4 ± 4.77	41.2 ± 5.82 *
CHOL, mg/dL	199 ± 13.8	201 ± 17.6	195.3 ± 9.80	204.3 ± 20.5
TG, mg/dL	123 ± 18.9	116 ± 18.4	173 ± 32.8	164.3 ± 15.44
ALB, g/dL	4.75 ± 0.12	4.09 ± 0.50	4.80 ± 0.32	4.20 ± 0.19
Total protein, g/dL	8.23 ± 0.34	6.44 ± 1.25	6.60 ± 0.5	7.36 ± 0.30
ALP, U/L	59.7 ± 10.1	43.7 ± 4.24	63.6 ± 11.2	64.6 ± 12.08
Ca, mg/dL	13.6 ± 4.00	9.92 ± 1.27	9.69 ± 0.87	11.3 ± 1.57
LDH, U/L	353 ± 21.3	358 ± 29.9	425 ± 23.5	406 ± 21.2
Mg, mg/dL	2.90 ± 0.55	2.14 ± 0.54	2.17 ± 0.33	2.45 ± 0.40
Pho, mg/dL	4.06 ± 0.20	3.9 ± 0.23	4.47 ± 0.40	3.95 ± 0.10
AST, U/L	22.3 ± 1.88	18.34 ± 2.37	23.49 ± 1.90	24.5 ± 3.57
ALT, U/L	21.6 ± 2.85	18.0 ± 4.00	19.29 ± 2.24	18.55 ± 2.17
Urea, mg/dL	30.4 ± 1.52	28.3 ± 2.58	38.2 ± 3.02	27.23 ± 3.29
ADMA, μmol/L	0.73 ± 0.08	0.93 ± 0.1	0.97 ± 0.09	1.23 ± 0.14 **
ADMA/L-arginine ratio	0.017 ± 0.002	0.021 ± 0.005	0.022 ± 0.002	0.026 ± 0.003

Results are shown as mean ± SEM, * *p* < 0.05, ** *p* < 0.01 vs. control. CRPhs, high-sensitive C-reactive protein; LDL, low density lipoproteins; HDL, high density lipoproteins; CHOL, total cholesterol; TG, triglycerides; ALB, albumin; Ca, calcium; ALP, alkaline phosphatase; LDH, lactate dehydrogenase; Mg, Magnesium; Pho, Phosphorus; AST, aspartate transaminase; ALT, alanine transaminase, ADMA, asymmetric dimethylarginine. * *p* < 0.05, ** *p* < 0.01 vs. control by one-way ANOVA followed by Holm-Sidak post hoc test.

**Table 2:** Characteristic of breast cancer patient subgroups.

Parameter	HR+ HER2+ (*n* = 12)	HR+ HER2- (*n* = 16)	TNBC (*n* = 19)
Histological type			
Ductal	8	13	16
Lobular	2	1	1
N.A.	2	2	2
Involved lymph nodes	2.00 ± 1.19	3.21 ± 1.53	2.69 ± 0.95
Ki, %	26.6 ± 3.39	14.1 ± 2.00 $	42.4 ± 5.35 ***
Hgb, g/dL	13.4 ± 0.46	13.6 ± 0.40	12.9 ± 0.32
Ht, %	39.1 ± 1.19	40.2 ± 1.09	38.1 ± 0.91
RBC, 10^6^/μL	4.64 ± 0.17	4.62 ± 0.15	4.20 ± 0.12
WBC, 10^3^/μL	7.25 ± 0.36	7.19 ± 0.35	6.86 ± 0.68
PLT, 10^3^/μL	245 ± 15.7	266 ± 22.6	291 ± 19.3
BUN mg/dL	12.3 ± 1.32	19.0 ± 3.93	15.7 ± 1.32
Creat, mg/dL	0.71 ± 0.04	0.94 ± 0.17	0.74 ± 0.04
Glu, mg/dL	103 ± 3.47	96.6 ± 3.17	106 ± 5.54
K, mmol/L	4.29 ± 0.13	4.52 ± 0.11	4.30 ± 0.09
Na mmol/L	139.3 ± 1.15	140 ± 0.71	139 ± 0.40
PT, s	11.3 ± 0.17	11.6 ± 0.20	11.4 ± 0.12
WPT	105.3 ± 1.81	102 ± 1.67	104 ± 1.14
INR	0.95 ± 0.02	0.98 ± 0.02	0.95 ± 0.01
APTT	30.3 ± 0.82	28.3 ± 0.91	28.3 ± 0.56
WAPPT	0.91 ±0.02	0.85 ± 0.03	0.85 ± 0.02
FIB, g/L	3.83 ± 0.46	3.32 ± 0.23	3.78 ± 0.70

Results are shown as mean ± SEM, *** *p* < 0.001 vs. HR+ HER2-, $ *p* < 0.05 vs. HR+ HER2+ by one-way ANOVA followed by Holm-Sidak post hoc test. N.A.—not available.

**Table 3:** Clinical correlations in TNBC patients with the activities of plasma ADA iso-enzymes.

	tADA		ADA1		ADA2	
Parameter	Correlation Coefficient	*p*-Value	Correlation Coefficient	*p*-Value	Correlation Coefficient	*p*-Value
Galectin-3, ng/mL	−0.08	NS	−0.28	NS	−0.37	NS
ADMA/L-arginine ratio	0.40	NS	0.54	0.03	0.42	NS
ADMA, μmol/L	0.51	0.03	0.73	0.0007	0.48	0.04
hsCRP, mg/dL	0.35	NS	0.63	0.008	0.05	NS

Results are shown as correlation plots and Pearson correlation coefficients with corresponded *p*-value.

## Data Availability

The data presented in this study are available on request from the corresponding author.

## Supplementary Materials

The following are available online at https://www.mdpi.com/article/10.3390/ijms22073764/s1, Table S1. Characteristic of hormone receptor positive and human epidermal growth factor positive breast cancer (HR+ HER2+ BC) patients at different stages of cancer development; Table S2. Characteristic of hormone receptor positive and human epidermal growth factor negative breast cancer (HR+ HER2- BC) patients at different stages of cancer development; Table S3. Characteristic of Triple Negative Breast Cancer (TNBC) patients at different stages of cancer development.

## Abbreviations

ADA	Adenosine deaminase
ADA1	Adenosine deaminase 1
ADA2	Adenosine deaminase 2
ADMA	Asymmetric dimethylarginine
Ado	Adenosine
ALB	Albumin
ALP	Alkaline phosphatase
ALT	Alanine transaminase
APTT	Activated partial thromboplastin time
AR	Adenosine receptor
AST	Aspartate transaminase
ATP	Adenosine triphosphate
BC	Breast cancer
BUN	Blood urea nitrogen
Ca	Calcium
CD39	Ecto-nucleoside triphosphate diphosphohydrolase
CD73	Ecto-5’nucleotidase
CHOL	Total cholesterol
CM	Cell medium
Creat	Creatinine
CRPhs	High sensitive C reactive protein
dAdo	Deoxyadenosine
dCF	Deoxycoformycin
eADA1	Ecto-adenosine deaminase 1
eADA2	Ecto-adenosine deaminase 2
EHNA	Erythro-9-(2-hydroxy-3-nonyl)adenine
ER	Estrogen receptors
FIB	Fibrinogen
Gal3	Galectin 3
Glu	Glucose
HDL	High density lipoproteins
HER2	Human epidermal growth factor 2
Hgb	Hemoglobin
HR	Hormone receptors
Ht	Hematocrit
HULEC	Human lung endothelial cell line
K	Potassium
Ki-67/Ki	Marker of proliferation Ki-67
LDH	Lactate dehydrogenase
LDL	Low density lipoproteins
M2	Macrophages M2
MDA-MB-231	Human triple negative breast cancer cell line
Mg	Magnesium
N.A.	Not available
Na	Sodium
Pho	Phosphorus
PLT	Platelets
PR	Progesterone receptors
PT	Prothrombin time
RBC	Red blood cells
RP-HPLC	Reverse phase high performance liquid chromatography
sCD163	Soluble CD163 protein
tADA	Total adenosine deaminase
TAMs	Tumor associated macrophages
TG	Triglycerydes
THP-1	Human monocytes/macrophages line
TILs	Tumor infiltrated lymphocytes
TNBC	Triple negative breast cancer
VEGF	Vascular endothelial growth factor
W APTT	Activated partial thromboplastin time factor
WBC	White blood cells
WPT	Prothrombin time factor
