# Supplementary material for: Macrophage-Derived Adenosine Deaminase 2 Correlates with M2 Macrophage Phenotype in Triple Negative Breast Cancer
Source: Int J Mol Sci. 2021 Apr 5;22(7):3764. doi: 10.3390/ijms22073764 (PMC8038600; doi:10.3390/ijms22073764)
Supplement: Supplementary file 1 [file ijms-22-03764-s001.pdf]

Table S1. Characteristic of hormone receptor positive and human epidermal growth factor positive breast cancer (HR+ HER2+ BC) patients at different stages of cancer development.

**Parameter**	**HR + HER2 + (*n* = 12)**		
Cancer stage	(I)<br>*n* = 3	(II)<br>*n* = 6	(III)<br>*n* = 3
Histological type			
Ductal	2	5	3
Lobular	0	1	0
N.A.	1	0	0
Involved lymph nodes	0 ± 0.00	0.80 ± 0.48	5.33 ± 3.53*
Ki, %	29.3 ± 6.36	25.5 ± 5.64	26.0 ± 4.00
Hgb, g/dl	13.25 ± 0.55	13.6 ± 0.49	12.5 ± 1.10
Ht, %	38.3 ± 2.00	39.7 ± 1.07	36.7 ± 2.98
RBC, 10^6/μl	4.50 ± 0.49	4.74 ± 0.15	4.24 ± 0.34
WBC, 10^3/μl	6.68 ± 0.92	7.23 ± 0.44	6.93 ± 1.01
PLT, 10^3/μl	245 ± 21.0	232 ± 16.8	321 ± 55.6
BUN mg/dl	9.80 ± 2.40	12.8 ± 1.68	10.5 ± 0.65
Creat, mg/dl	0.68 ± 0.01	0.69 ± 0.04	0.81 ± 0.07
Glu, mg/dl	91.5 ± 0.50	104 ± 4.23	105 ± 4.16
K, mmol/l	4.35 ± 0.05	4.52 ± 0.16	3.93 ± 0.17
Na mmol/l	139 ± 0.00	140 ± 1.69	139 ± 1.67
PT, s	11.5 ± 0.50	11.5 ± 0.22	11.3 ± 0.33
WPT	104 ± 7.50	104 ± 1.94	104 ± 3.48
INR	0.98 ± 0.06	0.96 ± 0.03	0.96 ± 0.01
APTT	31.5 ± 0.5	29.5 ± 1.06	29.0 ± 2.08
WAPPT	0.95 ± 0.01	0.88 ± 0.03	0.88 ± 0.06
FIB, g/l	3.49 ± 0.99	3.93 ± 0.57	3.20 ± 0.05

Results are shown as mean ± SEM, *$p < 0.05$, vs. (I) stage by one-way ANOVA followed by Holm-Sidak post hoc test. N.A. – not available.

Table S2. Characteristic of hormone receptor positive and human epidermal growth factor negative breast cancer (HR+ HER2- BC) patients at different stages of cancer development.

**Parameter**	**HR + HER2 - (*n* = 16)**		
Cancer stage	(I)<br>*n* = 7	(II)<br>*n* = 4	(III)<br>*n* = 4
Histological type			
Ductal	4	4	4
Lobular	1	0	0
N.A.	2	0	0
Involved lymph nodes	0 ± 0.00	2.33 ± 1.96**	1.00 ± 1.00*
Ki, %	13.7 ± 3.47	10.3 ± 2.72	17.6 ± 3.49
Hgb, g/dl	13.9 ± 0.21	12.8 ± 1.25	13.8 ± 0.46
Ht, %	41.0 ± 0.61	37.9 ± 3.77	40.7 ± 1.54
RBC, 10^6/μl	4.67 ± 0.14	4.45 ± 0.40	4.57 ± 0.23
WBC, 10^3/μl	6.88 ± 0.38	7.54 ± 0.68	7.28 ± 0.55
PLT, 10^3/μl	272 ± 22.9	242 ± 18.4	298 ± 43.6
BUN mg/dl	13.7 ± 1.39	29.7 ± 13.8	15.6 ± 1.74
Creat, mg/dl	0.69 ± 0.03	1.38 ± 0.6	0.82 ± 0.06
Glu, mg/dl	99.0 ± 10.8	102 ± 7.21	94.4 ± 3.60

K, mmol/l	4.38 ± 0.16	4.47 ± 0.18	4.62 ± 0.16
Na mmol/l	139 ± 0.67	140 ± 1.67	139 ± 1.24
PT, s	11.5 ± 0.22	11.7 ± 0.33	11.8 ± 0.37
WPT	96 ± 2.21	103 ± 4.10	101 ± 2.90
INR	0.97 ± 0.02	0.94 ± 0.03	0.98 ± 0.04
APTT	28.5 ± 0.76	27.3 ± 2.33	28.8 ± 1.42
WAPPT	0.85 ± 0.02	0.82 ± 0.07	0.86 ± 0.05
FIB, g/l	3.32 ± 0.32	3.65 ± 0.10	3.08 ± 0.42

Results are shown as mean ± SEM, *$p < 0.05$, **$p < 0.01$ vs. (I) stage by one-way ANOVA followed by Holm-Sidak post hoc test. N.A. – not available.

Table S3. Characteristic of Triple Negative Breast Cancer (TNBC) patients at different stages of cancer development.

**Parameter**	**Triple Negative Breast Cancer (*n* = 19)**		
Cancer stage	(I)<br>*n* = 7	(II)<br>*n* = 5	(III)<br>*n* = 7
Histological type			
Ductal	6	4	5
Lobular	0	1	0
N.A.	1	0	2
Involved lymph nodes	0	1.40 ± 1.16$	7.00 ± 1.78#
Ki, %	36.9 ± 7.53	39.6 ± 11.2	54.6 ± 11.7
Hgb, g/dl	13.1 ± 0.42	13.3 ± 0.47	12.2 ± 0.68
Ht, %	38.9 ± 1.30	39.2 ± 1.36	35.9 ± 1.95
RBC, 10^6/μl	4.30 ± 0.15	4.46 ± 0.21	3.79 ± 0.19
WBC, 10^3/μl	8.01 ± 1.60	6.14 ± 0.77	6.53 ± 1.23
PLT, 10^3/μl	262 ± 38.7	306 ± 36.8	301 ± 24.6
BUN mg/dl	20.1 ± 2.54	15.3 ± 2.06	12.5 ± 1.45
Creat, mg/dl	0.77 ± 0.04	0.75 ± 0.05	0.68 ± 0.08
Glu, mg/dl	106 ± 8.06	116 ± 12.1	94.2 ± 3.89
K, mmol/l	4.23 ± 0.12	4.34 ± 020	4.32 ± 0.14
Na mmol/l	138 ± 0.85	139 ± 0.50	140 ± 0.65
PT, s	11.1 ± 0.17	11.6 ± 0.20	11.5 ± 0.22
WPT	105 ± 1.43	104 ± 2.56	103 ± 1.80
INR	0.95 ± 0.02	0.94 ± 0.01	0.96 ± 0.01
APTT	28.6 ± 1.02	28.0 ± 0.53	28.3 ± 1.45
WAPPT	0.86 ± 0.03	0.84 ± 0.01	0.86 ± 0.05
FIB, g/l	4.02 ± 0.65	3.51 ± 0.30	3.85 ± 0.45

Results are shown as mean ± SEM, $$p < 0.05$ vs. (III) stage, #$p < 0.005$ vs. (I) stage one-way ANOVA followed by Holm-Sidak post hoc test. N.A. – not available.
